# The prognostic value of pretreatment neutrophil-lymphocyte ratio and platelet-lymphocyte ratio in patients with esophageal cancer undergoing immunotherapy: a systematic review and meta-analysis

**DOI:** 10.3389/fonc.2025.1536920

**Published:** 2025-02-14

**Authors:** Min Deng, Yun Qing, Dan Qiu, Ya Sheng, Juan Zhou, Lan Sun

**Affiliations:** Department of Oncology, Bishan Hospital of Chongqing Medical University, Chongqing, China

**Keywords:** esophageal cancer (EC), immunotherapy, neutrophil-to-lymphocyte ratio (NLR), platelet-to-lymphocyte ratio (PLR), prognosis, meta-analysis

## Abstract

**Background:**

Esophageal cancer (EC) is associated with a high morbidity and mortality rate. Immunotherapy has demonstrated effective antitumor activity in patients with EC, making it imperative to investigate easily accessible prognostic factors. Consequently, we conducted a meta-analysis to explore the prognostic significance of neutrophil-to-lymphocyte ratio (NLR) and platelet-to-lymphocyte ratio (PLR) in EC patients treated with immunotherapy.

**Methods:**

The literature search was conducted across three databases: PubMed, Embase, and Web of Science. The primary deadline for literature retrieval was July 2024. Hazard ratio (HR) with a 95% confidence interval (CI) was utilized to assess the association between NLR or PLR and overall survival (OS) as well as progression-free survival (PFS). Statistical analysis was performed using Review Manager version 5.4 and STATA version 15.0.

**Results:**

The meta-analysis included a total of 16 studies involving 1,481 patients. The results indicated a significant correlation between high pretreatment NLR and poor PFS (HR=1.76, 95%CI:1.38-2.25, p<0.001) as well as poor OS (HR=2.61,95%CI:1.86-3.67, p<0.001). Subgroup analyses based on tumor stage revealed that the association between elevated NLR and poor PFS was only observed in advanced EC patients. Regarding PLR, an increased PLR was found to be indicative of inferior PFS (HR=1.44, 95%CI: 1.20-1.72, p<0.001) and OS (HR=1.72,95%CI:1.08-2.74, p=0.020). However, the sensitivity analyses suggested that the observed increase in PLR lack robustness in terms of its impact on inferior OS.

**Conclusion:**

Elevated NLR and PLR are associated with inferior PFS and OS in EC patients receiving immunotherapy. These findings suggest that NLR and PLR levels hold promise as prognostic biomarkers in clinical practice, offering valuable guidance for personalized immunotherapy strategies.

**Systematic Review Registration:**

PROSPERO https://www.crd.york.ac.uk/prospero/, identifier CRD42024596737.

## 1 Introduction

The incidence of esophageal cancer (EC) ranks seventh, while its mortality rate ranks sixth in the world. Approximately 70% of cases occur in men. Eastern Asia exhibits the highest regional incidence rates, primarily due to the substantial burden in China (1). The predominant histopathological subtypes encompass esophageal squamous cell carcinoma (ESCC) and esophageal adenocarcinoma (EAC), with ESCC accounting for approximately 90% of annual cases (2).

The current treatment options encompass multimodality therapy, which comprises the mainstays of surgery, radiotherapy, chemotherapy, targeted therapy, and immunotherapy. The latest research findings demonstrated that immunotherapy has yielded substantial survival advantages for the patients diagnosed with EC (3–5), and ESCC was more sensitive to immunotherapy than EAC (6). Among EC patients who underwent resection after receiving neoadjuvant chemoradiotherapy, CheckMate577 demonstrated that those who received adjuvant therapy with nivolumab had a significantly longer disease-free survival (DFS) compared to those who received placebo (5). For patients with advanced EC, the combination of chemotherapy and immunotherapy offers a more significant survival advantage compared to chemotherapy alone. In the first-line treatment of advanced EC patients, the efficacy of immunotherapy and chemotherapy has been demonstrated in numerous phase III clinical trials. For example, KEYNOTE-590 found that pembrolizumab plus chemotherapy improved OS and PFS in patients with previously untreated, locally advanced, unresectable or metastatic EC (3). The CheckMate648 study found that the addition of nivolumab to chemotherapy as first-line treatment led to a significantly prolonged OS compared to chemotherapy alone (13.2 vs. 10.7 months; HR=0.74, 99.1% CI: 0.58 to 0.96; P = 0.002) in patients with advanced ESCC. Additionally, the combination of nivolumab and ipilimumab as first-line treatment also resulted in a significantly longer OS than chemotherapy alone (median, 12.7 vs. 10.7 months; hazard ratio, 0.78; 98.2% CI, 0.62 to 0.98; P = 0.01) (7). The studies of JUPITER-06, ORIENT-15 and ESCORT-1^st^ have also confirmed that the combination of toripalimab, sintilimab or camrelizumab with chemotherapy leads to significant benefits in OS and PFS (4, 8, 9). In the second-line treatment of advanced EC patients, KEYNOTE-181 revealed pembrolizumab prolonged OS compared to chemotherapy in patients with PD-L1 CPS ≥ 10, while also presenting a reduced incidence of treatment-related adverse events (10). The ESCORT trial and RATIONALE-302 trial demonstrated that second-line camrelizumab and tislelizumab improved OS in patients with advanced or metastatic ESCC compared to chemotherapy (11, 12). Furthermore, in patients with PD-L1 TAP ≥ 10%, tislelizumab demonstrated a statistically significant survival advantage over chemotherapy (12). Therefore, the utilization of immunotherapy is progressively increasing, necessitating the requirement for convenient and cost-effective indicators to assess the prognosis.

The neutrophil-to-lymphocyte ratio (NLR), a systemic inflammatory marker, is determined by the ratio of circulating neutrophil counts to lymphocyte counts. The Platelet-to-Lymphocyte Ratio (PLR) is a quantitative measure of systemic inflammation, obtained by dividing the circulating platelet count by the lymphocyte count. Previous studies have demonstrated the prognostic role of NLR and PLR in many malignant tumors, such as lung cancer, breast cancer and prostate cancer (13–16). The results of a meta-analysis have demonstrated that raised NLR and PLR are associated with unfavorable OS and PFS in advanced gastric cancer and gastroesophageal junction cancer patients undergoing immunotherapy (17). However, the prognostic significance of NLR and PLR in EC patients treated with immunotherapy remains controversial.

Therefore, we conducted a systematic review and meta-analysis to assess the prognostic roles of NLR and PLR in EC patients receiving immunotherapy.

## 2 Materials and methods

### 2.1 Search strategy

This systematic review and meta-analysis followed the PRISMA guidelines for the reporting of meta-analyses. The literature search was conducted across three databases: PubMed, Embase, and Web of Science. The primary deadline for literature retrieval was July 2024. The search strategy employed the following terms: (“Esophageal neoplasms” OR “Esophageal cancer” OR “Carcinoma, Esophagus”) AND (“NLR” OR “PLR” OR “neutrophil” OR “platelet”) AND (“immunotherapy” OR “PD” OR “checkpoint” OR “pembrolizumab” OR “nivolumab” OR “atezolizumab” OR “ipilimumab” OR “avelumab” OR “durvalumab” OR “camrelizumab” OR “tislelizumab” OR “Sintilimab”). The specific retrieval strategy is detailed in **Supplementary Text S1**.

This meta-analysis was registered in the PROSPERO network with the following ID: CRD42024596737.

### 2.2 Exclusion and inclusion criteria

The included studies met the following criteria: (a) patients with EC who received immunotherapy were included, regardless of treatment line; (b) investigation was conducted to determine the prognostic significance of baseline NLR or PLR in relation to OS or PFS; (c) the 95% confidence interval (CI) and hazard ratio (HR) could be obtained from the original studies; (d) publication in English literature was required.

The exclusion criteria were as follows: (a) systematic reviews, case reports, abstracts, letters, and expert opinions; (b) populations of patients with other primary tumors; (c) studies lacking sufficient data to conclude on the HR and 95% CI; (d) literature with Newcastle-Ottawa Scale (NOS) scores below 6; (e) non-English publications.

### 2.3 Literature’s data extraction and quality validation

Two authors independently extracted the following information from all eligible studies: The first author’s name, the year of publication, period of study, median follow-up (months), study design, country, sample size, pathological category, cut-off values, survival data (PFS or OS), and hazard ratio (HR) and 95% confidence interval (CI). The HRs from the multivariate analysis were initially extracted when both multivariate and univariate analyses were conducted. In this meta-analysis, we employed the median value of NLR or PLR cut-off from the studies included to determine subgroup analysis cut-off values.

We assessed the quality of the literature involved based on the scoring system of the Newcastle–Ottawa Scale (NOS) (18). The NOS encompasses three key components: patient selection, comparability, and outcome assessment. The studies rated 6 or higher were deemed to possess high quality. Studies with lower scores were considered low quality and thus excluded from the analysis.

### 2.4 Statistical analysis

HRs and their corresponding 95% CIs were pooled employing the generic inverse variance and random effects model. Heterogeneity was assessed using the Higgins I^2^ model. Significant heterogeneity was indicated when the values of I^2^≥50% and p<0.05, in which case a random-effects model was employed. Conversely, studies that did not exhibit significant heterogeneity were evaluated using a fixed-effects model. Subgroup analyses were also conducted to investigate potential factors influencing the prognostic significance of NLR and PLR. Subsequent sensitivity analyses were conducted to identify the sources of heterogeneity and evaluate the stability of the results. Egger’s test and funnel plots were conducted to assess potential publication bias, where a value of p<0.05 was considered a statistically significant difference. The statistical analysis was conducted using Review Manager version 5.4 and STATA version 15.0.

## 3 Results

### 3.1 Literature search and study characteristics

**Figure 1:**
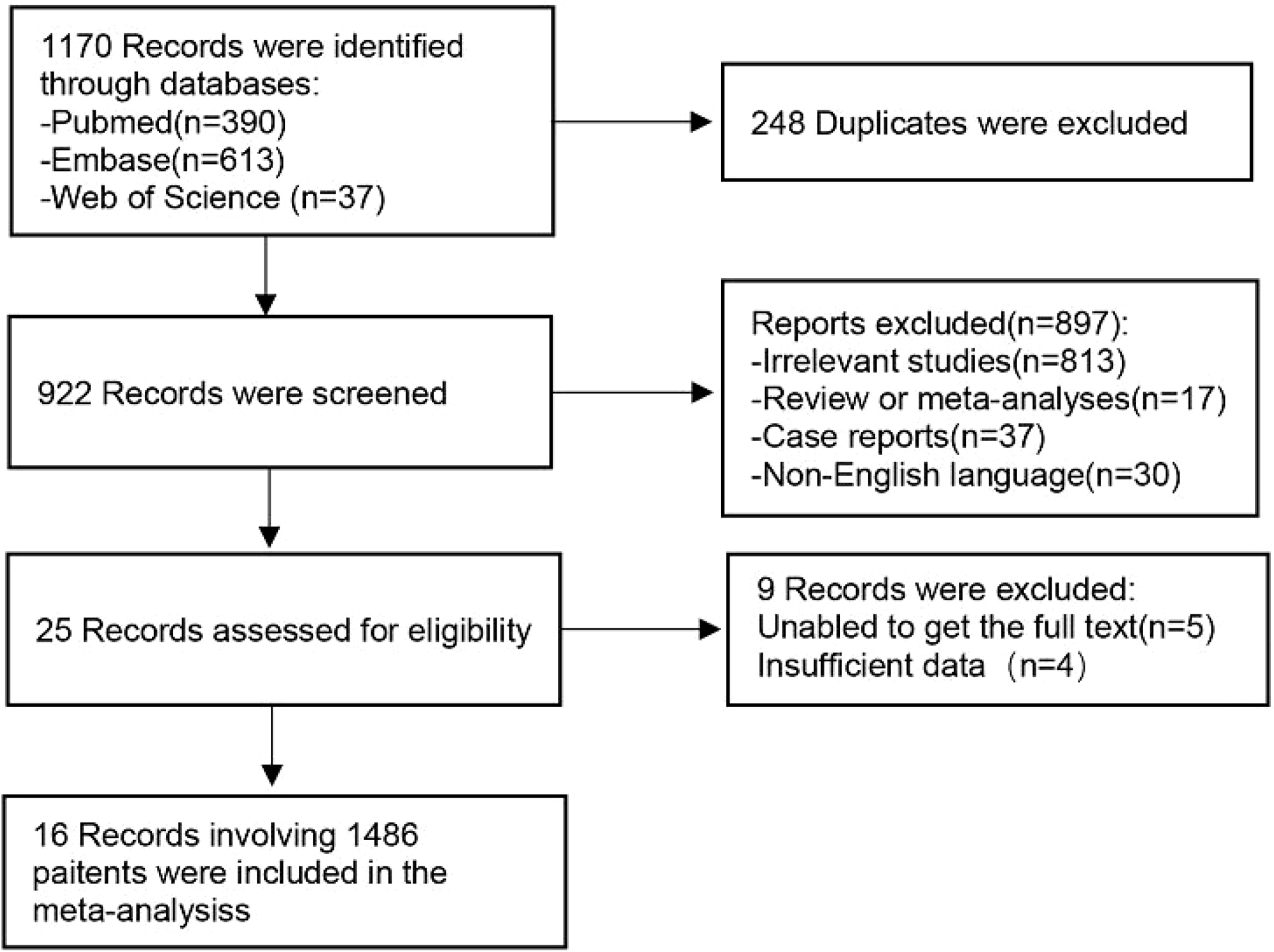
Flow chart of the study selection process.

A total of 1170 articles were retrieved from the three databases. After removing duplicate entries, a preliminary screening based on titles and abstracts was conducted for 922 articles, out of which 897 were deemed irrelevant to the subject matter under review. Subsequently, a thorough examination of the full texts of the remaining 25 studies was performed, resulting in the exclusion of 9 studies according to our predefined exclusion criteria. Ultimately, this systematic review comprised 16 selected studies (19–34). The selection process is summarized in **Figure 1**.

The primary characteristics of the studies included in the meta-analysis are presented in **Table 1**. In summary, the sample sizes of the cases ranged from 41 to 322 across these studies, which were all published between 2019 and 2024. 13 studies focused on patients diagnosed with ESCC, whereas the remaining 3 studies encompassed patients with various histological subtypes. The study conducted by Gao et al. analyzed data from 140 patients, with 130 (92.86%) diagnosed with squamous cell carcinoma, 4 (2.86%) with adenocarcinoma, and 6 (4.29%) with an unspecified histological subtype. Inoue et al.’s study included 41 patients, of whom 38 (92.68%) were diagnosed with squamous cell carcinoma. Sugase et al. examined data from 65 patients, comprising 62 cases (95.38%) of squamous cell carcinoma and 3 cases (4.62%) of adenocarcinoma. Among the included studies, 4 focused solely on NLR, while 12 evaluated both NLR and PLR. The meta-analysis comprised sixteen NLR studies with a total of 1481 cases and twelve PLR studies with a total of 1164 cases. All the studies were conducted in Asia, including 11 studies in China, 4 studies in Japan, and 1 study in Korea. The quality assessment using NOS scores revealed that the involved literature demonstrated high quality, with scores ranging from 7 to 9 among the sixteen studies. Detailed information regarding the quality assessment can be found in **Supplementary Table S1**.

**Table 1:** Included study of characteristics.

Study	Year	Study period	Median follow-up	country	Study design	Sample size	Median age(years)	Histology	stage	ICI	Biomarker	Cutoff method	NLR cut-off	PLR cut-off	Outcomes	Center disign
Chen et al. (19)	2023	Aug 2019- Aug 2021	Unclear	China	R	54	67	ESCC	advanced	Pembrolizumab,camrelizumab, sintilimab, tislelizumab	NLR,PLR	Literature	5	170.5	PFS	Single-center
Da et al. (20)	2023	Aug 2019- Feb 2022	16.9 months	China	R	162	66	ESCC	advanced	Camrelizumab, sintilimab, toripalimab	NLR,PLR	ROC	4.748	250.505	PFS,OS	Single-center
Gao et al. (21)	2022	Jan 2016-Mar 2020	20.0 months	China	R	140	60	EC	I-IV	Pembrolizumab,oripalizumab, Nivolumab, sintilimab, camrelizumab	NLR	Uncler	5	NR	PFS,OS	Single-center
GUO et al. (22)	2019	Aug 2015- Dec 2017	Unclear	China	R	49	56.7	ESCC	advanced	undisclosed	NLR	Median	6.4	NR	PFS,OS	Multi-center
Hamai et al. (23)	2023	Jun 2016- Dec 2021	Unclear	Japan	R	59	69.4	ESCC	advanced	Nivolumab	NLR	ROC	4.9	NR	PFS,OS	Single-center
Ikoma et al. (24)	2023	Jan 2017–Jun 2021	9.1 months	Japan	R	93	70	ESCC	advanced	Nivolumab	NLR,PLR	ROC	3.18	277	OS	Multi-center
INOUE et al. (25)	2022	Feb 2020- Apr 2022	294 days	Japan	R	41	68	EC	I-IV	Nivolumab	NLR,PLR	Median	3.401	242.6	PFS,OS	Single-center
Ji et al. (26)	2023	Oct 2016- Jul 2020	unclear	China	R	322	60	ESCC	Advanced	Camrelizumab, sintinimab	NLR,PLR	ROC	4	145	PFS,OS	Multi-center
Kim et al. (27)	2022	2015-2019	16.0 months	Korea	R	60	68	ESCC	Advanced	Nivolumab, Pembrolizumab	NLR,PLR	ROC	2.71	216.35	PFS,OS	Single-center
Liu et al. (28)	2022	Aug 2019- Oct 2021.	11.4 months	China	R	90	67	ESCC	Advanced	Camrelizumab	NLR,PLR	ROC	3.84	157.7	PFS,OS	Single-center
Qi et al. (29)	2023	Mar 2019-Mar 2022	20 months	China	R	51	62	ESCC	II-IVA	Pembrolizumab	NLR,PLR	ROC	2.6	150.63	PFS	Single-center
Shang et al. (30)	2024	Jul 2020- Jun 2022.	Unclear	China	R	64	63	ESCC	Advanced	Camrelizumab	NLR,PLR	ROC	4.6	194.5	PFS,OS	Single-center
Sugase et al. (31)	2024	Dec 2021- May 2023	11.6 months	Japan	P	65	65	EC	Advanced	Pembrolizumab	NLR,PLR	ROC	4.6	203	PFS,OS	Single-center
Wang et al. (33)	2022	May 2016- Jul 2018.	48.6 months	China	P	69	61	ESCC	Advanced	Camrelizumab	NLR	Literature	4	NR	PFS,OS	Single-center
Wang et al. (32)	2023	Oct 2019 -Oct 2021	Unclear	China	R	43	61	ESCC	II-IV	Pembrolizumab	NLR,PLR	Median	2.43	139.7	OS	Single-center
Wu et al. (34)	2021	Dec 2018 -Sep 2020	Unclear	China	R	119	61	ESCC	I-IV	Camrelizumab, nivolumab, pembrolizumab, toripalimab, sintilimab	NLR,PLR	Median	3.23	174.72	PFS	Single-center

(R, Retrospective; P, Prospective; PLR, platelet-to-lymphocyte ratio; OS, overall survival;).

### 3.2 Influence of NLR on PFS

**Figure 2:**
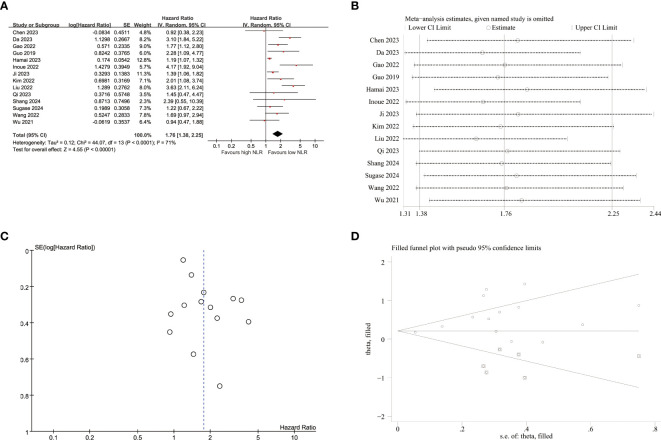
Pooled analyses of the association between pro-treatment NLR and PFS in EC patients. **(A)** Forest plot of the correlation between NLR and PFS. **(B)** Sensitivity analysis for PFS after excluding each study. **(C)** Funnel plot of publication bias regarding PFS. **(D)** Funnel plot adjusted by the trim and fill method regarding PFS.

14 studies reported the correlation between NLR and PFS (**Figure 2A**). The results indicated that elevated NLR was significantly associated with poor PFS outcomes (HR=1.76, 95%CI:1.38-2.25, p<0.001). Due to substantial heterogeneity observed among the included studies (I^2^ = 71%, p<0.001), a random-effects model was employed for meta-analysis. In the subgroup analyses of tumor stage, a significant association between elevated NLR and poor PFS was observed only in advanced EC. The subgroup analyses, as presented in **Table 2**, demonstrated no association between elevated PLR and unfavorable PFS within subgroups characterized by prospective study, and geographical origin from Japan.

**Table 2:** NLR subgroup analysis for PFS.

Variables	N	Effects model	PFS		Heterogeneity	
			HR (95%CI)	p-value	I^2^	p-value
Total	14	Random	1.76 (1.38, 2.25)	<0.001	71%	<0.001
Cut-off value						
<4	5	Random	2.17 (1.24, 3.81)	0.006	67%	0.020
≥4	9	Random	1.56 (1.24, 1.97)	<0.001	59%	0.010
Cut-off method						
ROC	8	Random	1.80 (1.30, 2.48)	<0.001	77%	<0.001
Not ROC	6	Random	1.72 (1.16, 2.53)	0.006	52%	0.060
Sample size						
<65	7	Random	1.76 (1.16, 2.66)	0.008	63%	0.010
≥65	7	Random	1.78 (1.28, 2.48)	<0.001	68%	0.005
Histology						
ESCC	11	Random	1.71 (1.30, 2.25)	<0.001	71%	<0.001
EC	3	Random	1.98 (1.08, 3.63)	0.030	67%	0.050
Stage						
Advanced	10	Random	1.75 (1.33, 2.32)	<0.001	73%	<0.001
I-IV	3	Random	1.87 (0.90, 3.87)	0.090	75%	0.020
II-IVA	1	–	1.45 (0.47, 4.47)	0.520	–	–
Survival analysis						
Multivariate	9	Random	1.80 (1.34, 2.42)	<0.001	76%	<0.001
Univariate	5	Random	1.68 (1.02, 2.78)	0.040	58%	0.050
Center						
Multicenter	2	Fixed	1.47 (1.14, 1.90)	0.003	34%	0.220
Single center	12	Random	1.79 (1.32, 2.43)	<0.001	74%	<0.001
Study design						
Retrospective	12	Random	1.83 (1.38, 2.43)	<0.001	75%	<0.001
Prospective	2	Fixed	1.45 (0.97, 2.18)	0.070	0%	0.430
Country						
China	10	Random	1.81 (1.36, 2.42)	<0.001	55%	0.020
Japan	3	Random	1.67 (0.89, 3.13)	<0.001	80%	0.007
Korea	1	–	2.01 (1.08, 3.74)	0.030	–	
Follow-up						
<12months	3	Random	2.60 (1.20, 5.64)	0.020	78%	0.010
≥12months	5	Fixed	2.03 (1.57, 2.63)	<0.001	0%	0.480
Unclear	6	Fixed	1.22 (1.11, 1.34)	<0.001	11%	0.350

(NLR, neutrophil-to-lymphocyte ratio; PFS, progression-free survival; HR, hazard ratio; CI, confidence interval).

Sensitivity analyses were conducted to assess the impact of individual studies on the relationship between NLR and PFS (**Figure 2B**; **Supplementary Table S2**). These analyses revealed that exclusion of any single study did not result in statistically significant changes regarding the influence of NLR on PFS outcomes. However, upon excluding Hamai et al.’s study, a notable decrease in heterogeneity was observed (I^2^ = 55%, p=0.009), yielding a pooled HR estimate of 1.86(95%CI:1.45-2.40, p<0.001).

### 3.3 NLR’s impact on OS

**Figure 3:**
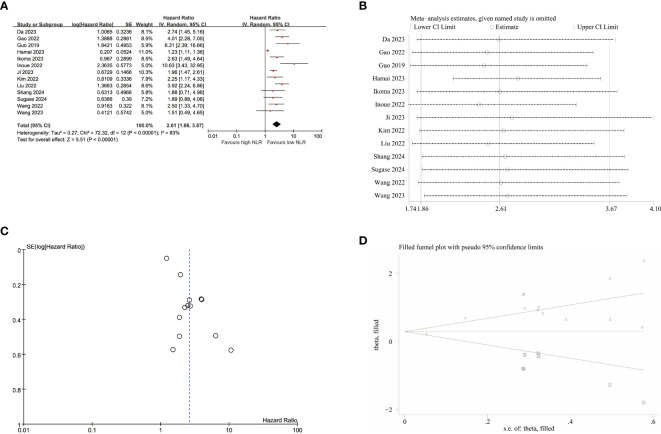
Pooled analyses of the association between pro-treatment NLR and OS in EC patients. **(A)** Forest plot of the correlation between NLR and OS. **(B)** Sensitivity analysis for OS after excluding each study. **(C)** Funnel plot of publication bias regarding OS. **(D)** Funnel plot adjusted by the trim and fill method regarding OS.

A total of 13 studies were included in the analysis to investigate the impact of NLR on OS, revealing high heterogeneity (I^2^ = 83%, p<0.001). Therefore, a random-effects model was employed, yielding a pooled hazard ratio (HR) of 2.61 (95% CI:1.86–3.67, p<0.001). These findings demonstrated that elevated NLR was significantly associated with worse OS in patients with EC (**Figure 3A**). Furthermore, apart from tumor stage II-IV, robust associations were confirmed between increased NLR and poor OS across all the subgroups, thus ensuring the reliability of our findings as presented in **Table 3**.

**Table 3:** NLR subgroup analysis for OS.

Variables	N	Effects model	OS		Heterogeneity	
			HR (95%CI)	p-value	I^2^	p-value
Total	13	Random	2.61 (1.86, 3.67)	<0.001	83%	<0.001
Cut-off value						
<4	5	Random	3.12 (1.95, 5.00)	<0.001	50%	0.090
≥4	8	Random	2.33 (1.58, 3.45)	<0.001	83%	<0.001
Cut-off method						
ROC	8	Random	2.14 (1.51, 3.03)	<0.001	81%	<0.001
Not ROC	5	Random	3.84 (2.23, 6.60)	<0.001	53%	0.070
Sample size						
<65	6	Random	2.62 (1.34, 5.13)	0.005	82%	<0.001
≥65	7	Fixed	2.49 (2.06, 3.01)	<0.001	30%	0.200
Histology						
ESCC	10	Random	2.33 (1.65, 3.29)	<0.001	81%	<0.001
EC	3	Random	3.96 (1.75, 8.97)	0.001	69%	0.040
Stage						
Advanced	10	Random	2.40 (1.71, 3.38)	<0.001	82%	<0.001
I-IV	2	Random	5.74 (2.28, 14.43)	<0.001	56%	0.130
II-IV	1	–	1.51 (0.49, 4.65)	0.47	–	–
Survival analysis						
Multivariate	9	Random	2.58 (1.74, 3.83)	<0.001	86%	<0.001
Univariate	4	Random	2.71 (1.33, 5.50)	<0.001	61%	0.050
Center						
Multicenter	3	Random	2.72 (1.59, 4.65)	<0.001	64%	0.060
Single center	10	Random	2.56 (1.65, 3.97)	<0.001	84%	<0.001
Study design						
Retrospective	11	Random	2.71 (1.84, 3.98)	<0.001	86%	<0.001
Prospective	2	Fixed	2.23 (1.37, 3.63)	0.001	0%	0.580
Country						
China	9	Fixed	2.55 (2.10, 3.09)	<0.001	44%	0.080
Japan	4	Random	2.46 (1.18, 5.13)	0.020	86%	<0.001
Korea	1	–	2.25 (1.17, 4.33)	0.020	–	–
Follow-up						
<12months	4	Random	3.40 (1.97, 5.88)	<0.001	58%	0.070
≥12months	4	Fixed	2.87 (2.11, 3.91)	<0.001	0%	0.550
Unclear	5	Random	1.89 (1.21, 2.95)	0.005	80%	<0.001

(NLR, neutrophil-to-lymphocyte ratio; OS, overall survival; HR, hazard ratio; CI, confidence interval).

Subsequently, sensitivity analyses were conducted to explore potential sources of heterogeneity for OS (**Figure 3B**; **Supplementary Table S3**), indicating that exclusion of any single study did not have a statistically significant impact for NLR’s influence on OS in this meta-analysis. After excluding the studies of Hamai et al, heterogeneity decreased to some extent (I^2^ = 43%, p=0.05), resulting in a merged HR of 2.79(95% CI:2.17-3.59, p<0.001).

### 3.4 Effect of PLR on PFS

**Figure 4:**
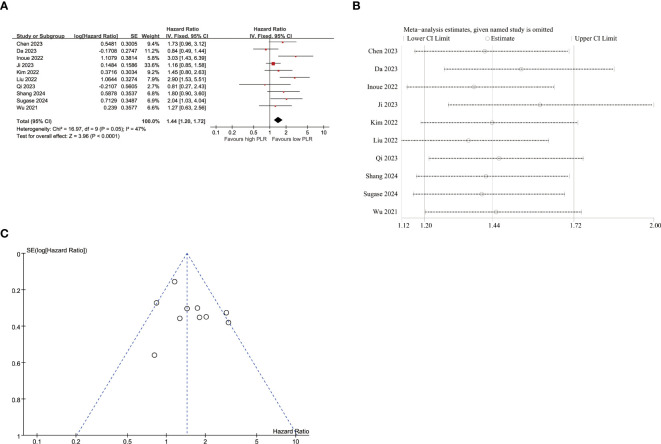
Pooled analyses of the association between pro-treatment PLR and PFS in EC patients. **(A)** Forest plot of the correlation between PLR and PFS. **(B)** Sensitivity analysis for PFS after excluding each study. **(C)** Funnel plot of publication bias regarding PFS.

10 studies were included in the analysis of the correlation between PFS and PLR (**Figure 4A**), employing a fixed-effects model due to low heterogeneity (I^2^ = 47%, p=0.050). The pooled results demonstrated a significant association between elevated PLR and poorer PFS (HR=1.44, 95% CI:1.20–1.72, p<0.001). As listed in **Table 4**, the subgroup analyses didn’t reveal the significant association between elevated PLR and poor PFS in subgroups with sample size≥65, tumor stage of I-IV and II-IV, multivariate survival analysis, multicenter studies, study conducted in Korea and follow-up duration≥12 months.

**Table 4:** PLR subgroup analysis for PFS.

Variables	N	Effects model	PFS		Heterogeneity	
			HR (95%CI)	p-value	I^2^	p-value
Total	10	Fixed	1.44 (1.20, 1.72)	<0.001	47%	0.050
Cut-off value						
<185	5	Fixed	1.38 (1.10, 1.75)	0.006	49%	0.100
≥185	5	Random	1.61 (1.05, 2.48)	0.030	55%	0.070
Cut-off method						
ROC	7	Random	1.43 (1.04, 1.97)	0.030	51%	0.060
Not ROC	3	Fixed	1.83 (1.24, 2.69)	0.002	29%	0.240
Sample size						
<65	5	Fixed	1.72 (1.26, 2.35)	<0.001	8%	0.360
≥65	5	Random	1.43 (0.96, 2.13)	0.080	63%	0.030
Histology						
ESCC	8	Fixed	1.33 (1.10, 1.61)	0.004	39%	0.120
EC	2	Fixed	2.44 (1.47, 4.04)	0.005	0%	0.440
Stage						
Advanced	7	Random	1.51 (1.13, 2.03)	0.006	50%	0.060
I-IV	2	Random	1.94 (0.83, 4.55)	0.130	64%	0.100
II-IV	1	–	0.81 (0.27, 2.43)	0.710	–	–
Survival analysis						
Multivariate	3	Random	1.36 (0.75, 2.47)	0.300	77%	0.010
Univariate	7	Fixed	1.69 (1.30, 2.20)	<0.001	0%	0.510
Center						
Multicenter	1	–	1.16 (0.85, 1.58)	0.350	–	–
Single center	9	Fixed	1.60 (1.29, 2.00)	<0.001	44%	0.080
Study design						
Retrospective	9	Random	1.49 (1.12, 1.98)	0.006	50%	0.040
Prospective	1	–	2.04 (1.03, 4.04)	0.040	–	–
Country						
China	7	Fixed	1.32 (1.08, 1.62)	0.008	48%	0.080
Japan	2	Fixed	2.44 (1.47, 4.04)	<0.001	0%	0.440
Korea	1	–	1.45 (0.80, 2.63)	0.220	–	–
Follow-up						
<12months	3	Fixed	2.61 (1.75, 3.88)	<0.001	0%	0.690
≥12months	3	Fixed	1.04 (0.72, 1.52)	0.830	0%	0.370
Unclear	4	Fixed	1.32 (1.04, 1.68)	0.020	0%	0.520

(PLR, platelet-to-lymphocyte ratio; PFS, progression-free survival; HR, hazard ratio; CI, confidence interval).

Sensitivity analyses were performed to evaluate the impact of individual studies on the association between PLR and PFS (**Figure 4B**; **Supplementary Table S4**). These analyses demonstrated that exclusion of any single study did not lead to statistically significant changes in terms of the influence of NLR on PFS outcomes.

### 3.5 PLR’s influence on OS

**Figure 5:**
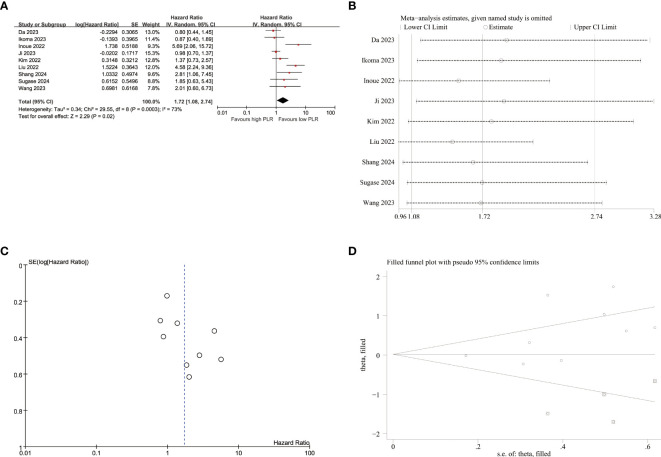
Pooled analyses of the association between pro-treatment PLR and OS in EC patients. **(A)** Forest plot of the correlation between PLR and OS. **(B)** Sensitivity analysis for OS after excluding each study. **(C)** Funnel plot of publication bias regarding OS. **(D)** Funnel plot adjusted by the trim and fill method regarding OS.

The data from 9 studies (**Figure 5A**) provided evidence on the impact of PLR on OS. A high degree of heterogeneity was observed among these studies (I^2^ = 73.00%, p<0.001). Consequently, a meta-analysis was conducted using the random-effect model, revealing a significant association between raised PLR and worse OS outcomes (HR=1.72,95%CI:1.08-2.74, p=0.020). The subgroup analyses, as presented in **Table 5**, demonstrated a significant correlation between elevated PLR and poor OS exclusively within subgroups characterized by cut-off method of not using receiver operating characteristic (ROC), sample size<65, histology of EC, tumor stage of I-IV, single-center study design, retrospective study, study conducted in China and duration of follow-up less than 12 months.

**Table 5:** PLR subgroup analysis for OS.

Variables	N	Effects model	OS		Heterogeneity	
			HR (95%CI)	p-value	I^2^	p-value
Total	9	Random	1.72 (1.08, 2.74)	0.020	73%	<0.001
Cut-off value						
<185	3	Random	2.02 (0.67, 6.11)	0.210	87%	<0.001
≥185	6	Random	1.60 (0.91, 2.81)	0.010	65%	0.010
Cut-off method						
ROC	7	Random	1.47 (0.92, 2.36)	0.110	71%	0.002
Not ROC	2	Fixed	3.70 (1.70, 8.05)	0.001	40%	0.200
Sample size						
<65	4	Fixed	2.17 (1.40, 3.35)	<0.001	48%	0.120
≥65	5	Random	1.36 (0.74, 2.51)	0.320	78%	0.001
Histology						
ESCC	7	Random	1.48 (0.92, 2.38)	0.110	72%	0.002
EC	2	Random	3.29 (1.10, 9.89)	0.030	55%	0.140
Stage						
Advanced	7	Random	1.47 (0.92, 2.36)	0.110	71%	0.002
I-IV	1	–	5.69 (2.06, 15.72)	<0.001	–	–
II-IV	1	–	2.01 (0.60, 6.73)	0.260		
Survival analysis						
Multivariate	5	Random	1.70 (0.87, 3.31)	0.120	80%	<0.001
Univariate	4	Random	1.80 (0.85, 3.79)	0.120	66%	0.030
Center						
Multicenter	2	Fixed	0.96 (0.71, 1.31)	0.800	0%	0.780
Single center	7	Random	2.17 (1.21, 3.87)	0.009	70%	0.003
Study design						
Retrospective	8	Random	1.72 (1.04, 2.85)	0.040	76%	<0.001
Prospective	1	–	1.85 (0.63, 5.43)	0.260	–	–
Country						
China	5	Random	1.72 (0.87, 3.39)	0.120	80%	<0.001
Japan	3	Random	2.02 (0.66, 6.18)	0.220	76%	0.020
Korea	1	–	1.37 (0.73, 2.57)	0.330	–	–
Follow-up						
<12months	4	Random	2.52 (1.02, 6.23)	0.040	76%	0.005
≥12months	2	Fixed	1.03 (0.67, 1.59)	0.890	33%	0.220
Unclear	3	Random	1.54 (0.74, 3.20)	0.250	59%	0.090

(PLR, platelet-to-lymphocyte ratio; OS, overall survival; HR, hazard ratio; CI, confidence interval).

Regarding the sensitivity analyses of OS (**Figure 5B**; **Supplementary Table S5**), exclusion of the study conducted by Liu et al. resulted in a partial decrease in heterogeneity (I^2^ = 59%, p=0.020). However, the combined hazard ratio (HR) didn’t maintain statistical significance upon exclusion of the study conducted by Liu et al. (HR=1.45, 95%CI:0.96-2.18, p=0.020) or Inoue et al. (HR=1.50, 95%CI: 0.97-2.33, p=0.070), suggesting that the observed increase in PLR may lack robustness in terms of its impact on inferior OS.

### 3.6 Publication bias

The funnel plots exhibited visual asymmetry in terms of the influence of NLR on PFS (**Figure 2C**) and OS (**Figure 3C**), suggesting a significant presence of publication bias. This observation was further substantiated by the results obtained from Egger’s test for PFS (p = 0.019) and OS (p < 0.001). Subsequently, trim and fill methods were employed to investigate the impact of publication bias on effect estimates. No statistically significant alterations were observed in the findings (**Figures 2D**, **3D**).

Regarding the influence of PLR on PFS (**Figure 4C**), the p-values of Egger’s test (p=0.255) did not indicate publication bias, as supported by symmetrical funnel plots upon visual inspection. The absence of publication bias detected by Egger’s test led us to refrain from employing additional trim and fill methods. In terms of the impact of PLR on OS (**Figure 5C**), the p-values of Egger’s test (p=0.022) revealed significant publication bias, which was evident from asymmetrical funnel plots upon visual examination. Furthermore, employing trim and fill methods once again demonstrated no statistically significant alterations in the results (**Figures 5D**).

## 4 Discussion

Esophageal cancer is a prevalent malignancy, and its management has been improved through continuous exploration of the disease and advancements in science and technology. Immune checkpoint inhibitors (ICIs) have demonstrated effective antitumor activity in patients with EC.

The utilization of immunotherapy in esophageal cancer is progressively expanding. However, the efficacy of immunotherapy may not be enhanced in all patients diagnosed with EC. Programmed cell death ligand 1 (PD-L1) and Tumor Mutational Burden (TMB) have emerged as biomarkers for predicting the efficacy of immunotherapy (35, 36). But their assessment requires complex and expensive laboratory techniques. Therefore, we need cost-effective, convenient, and rapid predictive biomarkers. Peripheral blood specimens, which exhibit high patient acceptance rates, are easier to obtain in clinical practice. Previous literatures have demonstrated that cancer-related inflammatory indicators, such as NLR and PLR, exhibit prognostic significance in patients with esophageal cancer undergoing immunotherapy (21, 22, 31).

The NLR, determined by the ratio of circulating neutrophil counts to lymphocyte counts, serves as a prognostic indicator for cancer patients (37, 38). Several studies have confirmed that neutrophils contribute to the processes of angiogenesis and immunosuppression (39–41). Coussens et al. revealed that the MMP-9 produced by neutrophils contributes to the carcinogenesis of squamous carcinogenesis (42). Christoffersson et al. demonstrated that the extracellular matrix is degraded by MMP-9 released from neutrophils, leading to the release of vascular endothelial growth factor (VEGF) and promotion of angiogenesis (43). Moreover, the release of Arg-1 from neutrophils leads to the downregulation of CD3ζ chain translation in T cells, thereby contributing to the inhibition of T cell proliferation. This mechanism establishes an immunosuppressive microenvironment that also facilitates cancer growth (44). Neutrophils also contribute to cancer progression by releasing cytokines and growth factors, such as IL-6, TNF, epidermal growth factor, hepatocyte growth factor (HGF), and platelet-derived growth factor (41, 45). The high PLR indicates an elevated platelet count or a reduced lymphocyte count, which may be indicative of tumor recurrence and metastasis. Platelets provides a procoagulant surface that enhances the amplification of cancer-related coagulation, and can be recruited to envelop tumor cells, thereby shielding them from immune responses and promoting cancer growth and dissemination (46). Tao et al. proposed that platelets have been implicated in inducing epigenetic modifications, such as upregulation of oncoproteins within circulating tumor cells, and secretion of potent growth factors may contribute to the promotion of mitogenesis, angiogenesis, and metastatic outgrowth (47). Additionally, numerous clinical and experimental studies have established the crucial role of lymphocytes in the immune response against tumors (48), and lymphopenia is correlated with an unfavorable prognosis in patients with recurrent metastatic EC patients who undergo immunotherapy (49).

In this present study, we conducted a meta-analysis by merging 16 studies on NLR involving 1481 EC cases and 12 studies on PLR involving 1164 EC cases to investigate the prognostic impact of NLR and PLR in patients treated with immunotherapy. The results of our meta-analysis revealed a significant association between elevated NLR and adverse PFS and OS outcomes. Additionally, the study by Wang et al. also corroborated that elevated pretreatment NLR is correlated with poorer outcomes in cancer immunotherapy (50). The prognostic effect of NLR with PFS remained robust in subgroup analyses considering various factors such as cut-off value, cut-off method, sample size, histology, survival analysis, research center and follow-up duration. Despite observing high heterogeneity, the association between increased NLR and poor OS was further supported by all subgroup analyses. These findings further support the reliability of our meta-analysis. Additionally, our combined findings regarding PLR demonstrated that increased PLR was also associated with unfavorable PFS and OS outcomes. This observation aligns with the study by Zhou et al., which demonstrated that lung cancer patients with lower PLR had superior OS and PFS when undergoing immunotherapy (51).The subgroup analyses didn’t reveal the significant association between elevated PLR and poor PFS in subgroups with sample size≥65, tumor stage of I-IV and II-IV, multivariate survival analysis, multicenter studies, study conducted in Korea and follow-up duration ≥12 months. Additionally, the subgroup analyses demonstrated a significant correlation between elevated PLR and poor OS exclusively within subgroups characterized by cut-off method of not using receiver operating characteristic (ROC), sample size<65, histology of EC, tumor stage of I-IV, single-center study design, retrospective study, study conducted in China and duration of follow-up less than 12 months. The combined hazard ratio (HR) didn’t remain significant upon exclusion of the study conducted by Liu et al. or Inoue et al. These findings suggest that the combined outcomes of PLR and OS may lack robustness, potentially due to the limited number of studies included in the meta-analysis. In the subgroup analyses of advanced EC patients undergoing immunotherapy, a significant association was observed between increased NLR and poor PFS as well as OS, while an elevated PLR was found to be linked with inferior PFS. Consistent with these findings, Matsas et al. reported that elevated NLR and PLR were also linked to unfavorable OS and PFS outcomes in patients with advanced gastric cancer (GC) and gastroesophageal junction cancer (GEJC) treated with immunotherapy (17).

As a literature-dependent meta-analysis, several limitations of this study should be acknowledged. Firstly, the majority of included studies were retrospective with small sample sizes, potentially introducing selection bias and influencing the findings. Therefore, it is imperative to conduct more large-scale prospective studies to validate our results. Secondly, only English-language publications were considered in this analysis, excluding non-English studies and unpublished data which may have limited the available evidence for analysis. Thirdly, variations in characteristics such as cut-off value, sample size, tumor stage, survival analysis, and follow-up duration across different studies could contribute to substantial heterogeneity observed in the meta-analysis. Fourthly, we can’t disregard the possibility that non-tumor-related factors might impact patients’ blood markers. Lastly but importantly, there was a lack of standardized cut-off values for NLR or PLR among included studies in this meta-analysis. The range of NLR cut-off values varied from 2.43 to 6.40 while PLR cut-off values ranged from 139.7 to 277 across different studies involved herein, thus limiting clinical applicability and necessitating standardization efforts for NLR and PLR thresholds.

## 5 Conclusion

Despite its limitations, our meta-analysis found the association between elevated peripheral blood NLR or PLR and inferior PFS and OS in EC patients receiving immunotherapy. These findings suggest that NLR and PLR levels hold promise as prognostic biomarkers in clinical practice, offering valuable guidance for personalized immunotherapy strategies. Future investigations should focus on prospective, multi-center, large-scale studies to validate the results of this meta-analysis and facilitate its integration with other prognostic indicators.

## Data Availability

The original contributions presented in the study are included in the article/**Supplementary Material**. Further inquiries can be directed to the corresponding author.
