# Supplementary figures and images for: The prognostic value of pretreatment neutrophil-lymphocyte ratio and platelet-lymphocyte ratio in patients with esophageal cancer undergoing immunotherapy: a systematic review and meta-analysis
Source: Front Oncol. 2025 Feb 14;15:1536920. doi: 10.3389/fonc.2025.1536920 (PMC11868166; doi:10.3389/fonc.2025.1536920)

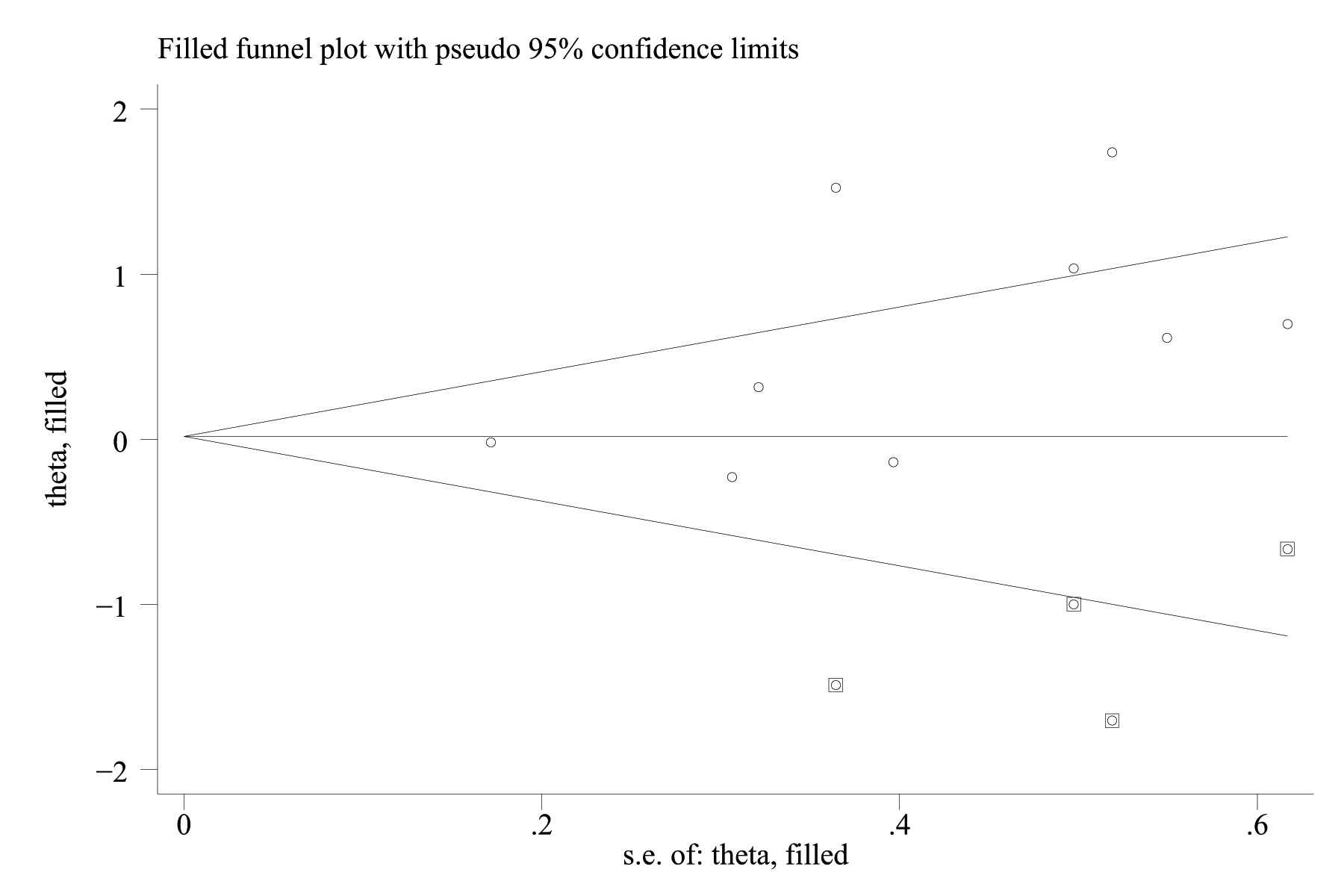

Supplement: Supplementary file 1 [file Image1.tif]
