# Supplementary material for: The prognostic value of pretreatment neutrophil-lymphocyte ratio and platelet-lymphocyte ratio in patients with esophageal cancer undergoing immunotherapy: a systematic review and meta-analysis
Source: Front Oncol. 2025 Feb 14;15:1536920. doi: 10.3389/fonc.2025.1536920 (PMC11868166; doi:10.3389/fonc.2025.1536920)
Supplement: Supplementary file 3 [file DataSheet1.zip › Supplementary Table S1.DOCX]

Supplementary Table S1. Newcastle–Ottawa Scale for quality assessment.

| Authors | Year | Selection | Comparability | Outcome | Total score |
| --- | --- | --- | --- | --- | --- |
| Chen et al. | 2023 | 4 | 2 | 2 | 7 |
| Da et al. | 2023 | 4 | 2 | 3 | 9 |
| Gao et al. | 2022 | 4 | 1 | 3 | 8 |
| Guo et al. | 2019 | 4 | 1 | 2 | 8 |
| Hamai et al. | 2023 | 4 | 1 | 2 | 7 |
| Ikoma et al. | 2023 | 4 | 2 | 2 | 8 |
| Inoue et al. | 2022 | 4 | 1 | 2 | 7 |
| Ji et al. | 2023 | 4 | 1 | 2 | 7 |
| Kim et al. | 2022 | 4 | 1 | 3 | 8 |
| Liu et al. | 2022 | 4 | 2 | 2 | 8 |
| Qi et al. | 2023 | 4 | 2 | 3 | 9 |
| Shang et al. | 2024 | 4 | 2 | 2 | 8 |
| Sugase et al. | 2024 | 4 | 2 | 2 | 8 |
| Wang et al. | 2022 | 4 | 1 | 2 | 7 |
| Wang et al. | 2023 | 4 | 2 | 3 | 9 |
| Wu et al. | 2021 | 4 | 2 | 2 | 8 |
