# Supplementary material for: The prognostic value of pretreatment neutrophil-lymphocyte ratio and platelet-lymphocyte ratio in patients with esophageal cancer undergoing immunotherapy: a systematic review and meta-analysis
Source: Front Oncol. 2025 Feb 14;15:1536920. doi: 10.3389/fonc.2025.1536920 (PMC11868166; doi:10.3389/fonc.2025.1536920)
Supplement: Supplementary file 3 [file DataSheet1.zip › Supplementary Table S2.DOCX]

**Supplementary Table S2.** Sensitivity analysis of the relationship between NLR and PFS.

| **Study omitted** | **HR (95% CI)** | ***P*-value** | **I^2^** | ***P*_H_** |
| --- | --- | --- | --- | --- |
| Chen et al. 2023 | 1.82 (1.41, 2.35) | < 0.001 | 72% | <0.001 |
| Da et al. 2023 | 1.66 (1.31, 2.10) | < 0.001 | 65% | <0.001 |
| Gao et al. 2022 | 1.76 (1.36, 2.29) | < 0.001 | 72% | <0.001 |
| Guo et al. 2019 | 1.73 (1.35, 2.23) | < 0.001 | 72% | <0.001 |
| Hamai et al. 2023 | 1.86 (1.45, 2.40) | < 0.001 | 55% | 0.009 |
| Inoue et al. 2022 | 1.67 (1.32, 2.10) | < 0.001 | 67% | <0.001 |
| Ji et al. 2023 | 1.82 (1.37, 2.44) | < 0.001 | 73% | <0.001 |
| Kim et al. 2022 | 1.74 (1.35, 2.25) | < 0.001 | 72% | <0.001 |
| Liu et al. 2022 | 1.63 (1.31, 2.05) | < 0.001 | 61% | 0.002 |
| Qi et al. 2023 | 1.77 (1.38, 2.28) | < 0.001 | 73% | <0.001 |
| Shang et al. 2024 | 1.75 (1.36, 2.24) | < 0.001 | 72% | <0.001 |
| Sugase et al. 2024 | 1.82 (1.40, 2.36) | < 0.001 | 73% | <0.001 |
| Wang et al. 2022 | 1.77 (1.36, 2.30) | < 0.001 | 72% | <0.001 |
| Wu et al. 2021 | 1.84 (1.43, 2.37) | < 0.001 | 72% | <0.001 |

Abbreviations: NLR, neutrophil-lymphocyte ratio; PFS, progression-free survival; HR, hazard ratio; CI, confidence interval; *P*_H_, *P-*value for heterogeneity.
