# Supplementary material for: The prognostic value of pretreatment neutrophil-lymphocyte ratio and platelet-lymphocyte ratio in patients with esophageal cancer undergoing immunotherapy: a systematic review and meta-analysis
Source: Front Oncol. 2025 Feb 14;15:1536920. doi: 10.3389/fonc.2025.1536920 (PMC11868166; doi:10.3389/fonc.2025.1536920)
Supplement: Supplementary file 3 [file DataSheet1.zip › Supplementary Table S3.DOCX]

**Supplementary Table S3.** Sensitivity analysis of the relationship between NLR and OS.

| **Study omitted** | **HR (95% CI)** | ***P*-value** | **I^2^** | ***P*_H_** |
| --- | --- | --- | --- | --- |
| Da et al. 2023 | 2.60 (1.82, 3.73) | < 0.001 | 84% | <0.001 |
| Gao et al. 2022 | 2.49 (1.77, 3.51) | < 0.001 | 82% | <0.001 |
| Guo et al. 2019 | 2.46 (1.76, 3.45) | < 0.001 | 83% | <0.001 |
| Hamai et al. 2023 | 2.79 (2.17, 3.59) | < 0.001 | 43% | 0.050 |
| Ikoma et al. 2023 | 2.61 (1.82, 3.75) | < 0.001 | 84% | < 0.001 |
| Inoue et al. 2022 | 2.41 (1.74, 3.35) | < 0.001 | 82% | <0.001 |
| Ji et al. 2023 | 2.73 (1.82, 4.10) | < 0.001 | 84% | <0.001 |
| Kim et al. 2022 | 2.65 (1.84, 3.81) | < 0.001 | 84% | <0.001 |
| Liu et al. 2022 | 2.50 (1.77, 3.53) | < 0.001 | 82% | <0.001 |
| Shang et al. 2024 | 2.67 (1.87, 3.82) | < 0.001 | 85% | <0.001 |
| Sugase et al. 2024 | 2.68 (1.87, 3.85) | < 0.001 | 85% | <0.001 |
| Wang et al. 2022 | 2.63 (1.83, 3.77) | < 0.001 | 84% | <0.001 |
| Wang et al. 2023 | 2.69 (1.89, 3.84) | < 0.001 | 85% | <0.001 |

Abbreviations: NLR, neutrophil-lymphocyte ratio; OS, Overall survival; HR, hazard ratio; CI, confidence interval; *P*_H_, *P-*value for heterogeneity.
