# Supplementary material for: The prognostic value of pretreatment neutrophil-lymphocyte ratio and platelet-lymphocyte ratio in patients with esophageal cancer undergoing immunotherapy: a systematic review and meta-analysis
Source: Front Oncol. 2025 Feb 14;15:1536920. doi: 10.3389/fonc.2025.1536920 (PMC11868166; doi:10.3389/fonc.2025.1536920)
Supplement: Supplementary file 3 [file DataSheet1.zip › Supplementary Table S4.DOCX]

**Supplementary Table S4.** Sensitivity analysis of the relationship between PLR and PFS.

| **Study omitted** | **HR (95% CI)** | ***P*-value** | **I^2^** | ***P*_H_** |
| --- | --- | --- | --- | --- |
| Chen et al. 2023 | 1.41 (1.17, 2.04) | <0.001 | 52% | 0.040 |
| Da et al. 2023 | 1.54 (1.27, 1.86) | <0.001 | 37% | 0.120 |
| Inoue et al. 2022 | 1.37 (1.14, 1.65) | <0.001 | 38% | 0.110 |
| Ji et al. 2023 | 1.60 (1.29, 2.00) | <0.001 | 44% | 0.080 |
| Kim et al. 2022 | 1.44 (1.19, 1.74) | <0.001 | 53% | 0.030 |
| Liu et al. 2022 | 1.35 (1.12, 1.63) | 0.002 | 33% | 0.150 |
| Qi et al. 2023 | 1.46 (1.22, 1.75) | <0.001 | 50% | 0.040 |
| Shang et al. 2024 | 1.42 (1.17, 1.71) | <0.001 | 52% | 0.040 |
| Sugase et al. 2024 | 1.40 (1.16, 1.69) | <0.001 | 50% | 0.040 |
| Wu et al. 2021 | 1.45 (1.20, 1.75) | <0.001 | 52% | 0.030 |

Abbreviations: PLR, platelet-lymphocyte ratio; PFS, progression-free survival; HR, hazard ratio; CI, confidence interval; *P*_H_, *P-*value for heterogeneity.
