# Supplementary material for: The prognostic value of pretreatment neutrophil-lymphocyte ratio and platelet-lymphocyte ratio in patients with esophageal cancer undergoing immunotherapy: a systematic review and meta-analysis
Source: Front Oncol. 2025 Feb 14;15:1536920. doi: 10.3389/fonc.2025.1536920 (PMC11868166; doi:10.3389/fonc.2025.1536920)
Supplement: Supplementary file 3 [file DataSheet1.zip › Supplementary Table S5.DOCX]

**Supplementary Table S5.** Sensitivity analysis of the relationship between PLR and OS.

| **Study omitted** | **HR (95% CI)** | ***P*-value** | **I^2^** | ***P*_H_** |
| --- | --- | --- | --- | --- |
| Da et al. 2023 | 1.94 (1.16, 3.24) | 0.010 | 73% | <0.001 |
| Ikoma et al. 2023 | 1.89 (1.13, 3.16) | 0.020 | 75% | <0.001 |
| Inoue et al. 2022 | 1.50 (0.97, 2.33) | 0.070 | 68% | 0.003 |
| Ji et al. 2023 | 1.92 (1.12, 3.28) | 0.020 | 70% | 0.001 |
| Kim et al. 2022 | 1.80 (1.05, 3.10) | 0.030 | 76% | <0.001 |
| Liu et al. 2022 | 1.45 (0.96, 2.18) | 0.080 | 59% | 0.020 |
| Shang et al. 2024 | 1.64 (1.00, 2.68) | 0.050 | 74% | <0.001 |
| Sugase et al. 2024 | 1.72 (1.04, 2.85) | 0.040 | 76% | <0.001 |
| Wang et al. 2023 | 1.71 (1.04, 2.81) | 0.040 | 76% | <0.001 |

Abbreviations: PLR, platelet-lymphocyte ratio; OS, overall survival; HR, hazard ratio; CI, confidence interval; *P*_H_, *P-*value for heterogeneity.
