# Supplementary material for: The prognostic value of pretreatment neutrophil-lymphocyte ratio and platelet-lymphocyte ratio in patients with esophageal cancer undergoing immunotherapy: a systematic review and meta-analysis
Source: Front Oncol. 2025 Feb 14;15:1536920. doi: 10.3389/fonc.2025.1536920 (PMC11868166; doi:10.3389/fonc.2025.1536920)
Supplement: Supplementary file 3 [file DataSheet1.zip › Supplementary Text S1.DOCX]

**Supplementary Text S1. The specific retrieval strategy in each database.**

1. **PubMed retrieval strategy**

(((((((((((((immunotherapy) OR (PD)) OR (checkpoint)) OR (nivolumab)) OR (pembrolizumab)) OR (ipilimumab)) OR (atezolizumab)) OR (avelumab)) OR (durvalumab)) OR (camrelizumab)) OR (tislelizumab)) OR (Sintilimab)) AND (((((plr) OR (nlr)) OR (platelet)) OR (neutrophil)))) AND ((((((((((((((((((Esophageal Neoplasms) OR (Esophageal Neoplasm)) OR (Neoplasm, Esophageal)) OR (Esophagus Neoplasm)) OR (Esophagus Neoplasms)) OR (Neoplasm, Esophagus)) OR (Neoplasms, Esophagus)) OR (Neoplasms, Esophageal)) OR (Cancer of Esophagus)) OR (Esophageal Cancer)) OR (Cancer, Esophageal)) OR (Cancers, Esophageal)) OR (Esophageal Cancers)) OR (Cancer of the Esophagus)) OR (Esophagus Cancer)) OR (Cancer, Esophagus)) OR (Cancers, Esophagus)) OR (Esophagus Cancers))

**(2) Embase retrieval strategy**

(plr OR nlr OR platelet OR neutrophil) AND ('immunotherapy'/exp OR immunotherapy OR pd OR checkpoint OR nivolumab OR pembrolizumab OR ipilimumab OR atezolizumab OR avelumab OR durvalumab OR camrelizumab OR tislelizumab OR sintilimab) AND ('esophageal neoplasms'/exp OR 'esophageal neoplasms' OR (esophageal AND ('neoplasms'/exp OR neoplasms)) OR (esophageal AND neoplasm) OR (neoplasm, AND esophageal) OR (esophagus AND neoplasm) OR (esophagus AND neoplasms) OR (neoplasm, AND esophagus) OR (neoplasms, AND esophagus) OR (neoplasms, AND esophageal) OR (cancer AND of AND esophagus) OR (esophageal AND cancer) OR (cancer, AND esophageal) OR (cancers, AND esophageal) OR (esophageal AND cancers) OR (cancer AND of AND the AND esophagus) OR (esophagus AND cancer) OR (cancer, AND esophagus) OR (cancers, AND esophagus) OR (esophagus AND cancers))

**(3) Web of Science retrieval strategy**

(plr (Topic) or nlr (Topic) or platelet (Topic) or neutrophil (Topic))and (immunotherapy (Topic) or PD (Topic) or checkpoint (Topic) or nivolumab (Topic) or pembrolizumab (Topic) or ipilimumab (Topic) or atezolizumab (Topic) or avelumab (Topic) or durvalumab (Topic) or camrelizumab (Topic) or tislelizumab (Topic) or Sintilimab (Topic) )and(Esophageal Neoplasms (Topic) or Esophageal Neoplasm (Topic) or Neoplasm, Esophageal (Topic) or Esophagus Neoplasm (Topic) or Esophagus Neoplasms (Topic) or Neoplasm, Esophagus (Topic) or Neoplasms, Esophagus (Topic) or Neoplasms, Esophageal (Topic) or Cancer of Esophagus (Topic) or Esophageal Cancer (Topic) or Cancer, Esophageal (Topic) or Cancers, Esophageal (Topic) or Esophageal Cancers (Topic) or Cancer of the Esophagus (Topic) or Esophagus Cancer (Topic) or Cancer, Esophagus (Topic) or Cancers, Esophagus (Topic) or Esophagus Cancers (Topic))
